# Effects of the auxin-dependent degradation of the cohesin and condensin complexes on the repair of distant DNA double-strand breaks in mouse embryonic stem cells

**DOI:** 10.18699/vjgb-24-65

**Published:** 2024-10

**Authors:** A.V. Smirnov, A.S. Ryzhkova, A.M. Yunusova

**Affiliations:** Institute of Cytology and Genetics of the Siberian Branch of the Russian Academy of Sciences, Novosibirsk, Russia; Institute of Cytology and Genetics of the Siberian Branch of the Russian Academy of Sciences, Novosibirsk, Russia; Institute of Cytology and Genetics of the Siberian Branch of the Russian Academy of Sciences, Novosibirsk, Russia

**Keywords:** CRISPR/Cas9, mouse embryonic stem cells, auxin, cohesin, condensin, DNA repair, CRISPR/Cas9, эмбриональные стволовые клетки мыши, ауксин, когезин, конденсин, репарация ДНК

## Abstract

The SMC protein family, including cohesin and condensin I/II, plays a pivotal role in maintaining the topological structure of chromosomes and influences many cellular processes, notably the repair of double-stranded DNA breaks (DSBs). The cohesin complex impacts DSB repair by spreading γH2AX signal and containing DNA ends in close proximity by loop extrusion. Cohesin supports DNA stability by sister chromatid cohesion during the S/G2 phase, which limits DNA end mobility. Cohesin knockdown was recently shown to stimulate frequencies of genomic deletions produced by distant paired DSBs, but does not affect DNA repair of a single or close DSBs. We examined how auxin-inducible protein degradation of Rad21 (cohesin) or Smc2 (condensins I+II) changes the frequencies of rearrangements between paired distant DSBs in mouse embryonic stem cells (mESCs). We used Cas9 RNP nucleofection to generate deletions and inversions with high efficiency without additional selection. We determined optimal Neon settings and deletion appearance timings. Two strategies for auxin addition were tested (4 independent experiments in total). We examined deletion/inversion frequencies for two regions spanning 3.5 and 3.9 kbp in size. Contrary to expectations, in our setting, Rad21 depletion did not increase deletion/inversion frequencies, not even for the region with an active Ctcf boundary. We actually observed a 12 % decrease in deletions (but not inversions). At the same time, double condensin depletion (Smc2 degron line) demonstrated high biological variability between experiments, complicating the analysis, and requires additional examination in the future. TIDE analysis revealed that editing frequency was consistent (30–50 %) for most experiments with a minor decrease after auxin addition. In the end, we discuss the Neon/ddPCR method for deletion generation and detection in mESCs.

## Abstract (ru)

Семейство SMC-белков, включающее когезин и конденсины I/II, играет ключевую роль в формировании топологической структуры хромосом и косвенно влияет на широкий спектр клеточных процессов, в том числе и на репарацию двуцепочечных разрывов ДНК (DSB). Комплекс когезина регулирует репарацию DSB на нескольких уровнях, например, распространяя сигнал γH2AX и удерживая концы ДНК в непосредственной близости за счет экструзии петель возле разрыва. Когезин также скрепляет сестринские хроматиды во время фазы S/G2, что ограничивает потенциальную подвижность концов ДНК. По имеющимся данным, в фибробластах человека нокдаун когезина стимулирует образование геномных делеций между удаленными DSB (3.2 тыс. п. о.), но не влияет на репарацию одиночных или близких DSB (34 п. о.). Мы решили проверить это наблюдение на эмбриональных стволовых клетках мыши, несущих ауксин-индуцибельный дегрон Rad21 (субъединица когезина) или Smc2 (субъединица конденсинов I+II). Для этого мы использовали нуклеофекцию RNP Cas9 и пары гайдовых РНК для генерации делеций и инверсий с высокой эффективностью без дополнительной селекции. Мы определили оптимальные условия для эффективной электропорации, включая настройки Neon, а также тайминги появления делеций. Были протестированы две стратегии добавления ауксина (суммарно четыре независимых эксперимента). Были исследованы частоты перестроек в двух сайтах размером около 3.5 и 3.9 тыс. п. о. Вопреки ожиданиям, деплеция Rad21 не увеличивала частоту делеций/инверсий, даже для региона с активной границей Ctcf. Фактически наблюдалось снижение частоты делеций (но не инверсий) на 12 %. Деплеция Smc2 не приводила к заметному увеличению частот делеций/инверсий, возможно, из-за высокой биологической изменчивости между экспериментами. Анализ TIDE показал, что частота редактирования была постоянной для большинства экспериментов (30–50 %), с незначительным снижением после добавления ауксина. В статье также обсуждается применимость метода Neon/ddPCR для создания и детекции делеций в эмбриональных стволовых клетках мыши.

## Introduction

Properly joining the two ends of a double-strand break (DSB) is crucial for preserving genome integrity. Unprocessed DNA ends can degrade, leading to loss of genetic information. Moreover, because DNA repair occurs in the vast space of the nucleus, incorrect ligation of multiple DSBs can result in chromosomal rearrangements, such as translocations, inversions, deletions, mitotic bridges and even chromothripsis. One-sided breaks that arise during replication are also highly dangerous and must be restrained and connected to the appropriate DNA molecule.

SMC complexes (cohesin, condensin-I, condensin-II) consist of several proteins organized into a ring-shaped structure (Kabirova et al., 2023). They utilize ATP-driven motor activity to shape and organize the genome into topological domains (TADs). Cohesin is an integral part of cellular homeostasis, regulating DNA conformation and topology, thus governing most vital processes from replication and cell division to gene expression and programmed DNA breaks in meiosis or V(D)J recombination. Although many reports have linked cohesin to DNA repair, its exact role therein remains unclear. Cohesin is attracted to DSB foci (Ström et al., 2004; Ünal et al., 2004), but is probably not essential for DNA end-joining per se (Gelot et al., 2016). Early cytogenetic and microscopic evidence suggests that cohesin limits DNA end mobility and prevents genomic rearrangements (Wu, Yu, 2012). A study using a genetic reporter showed that cohesin knockdown leads to an increased frequency of deletions when two DSBs are introduced at a distance of 3.2 kbp but does not affect the ligation of closely located breaks (34 bp) (Gelot et al., 2016).

Importantly, these observations were only relevant to the S phase, where cohesin is required for sister chromatid cohesion. Knockdown of cohesin in G1-synchronized cells did not have an effect on deletion frequencies, probably because cohesin molecules physically limit the mobility of the DSB ends to preserve genome integrity only during S phase (Supplementary Material 1)1. Generally speaking, cohesin removal does not affect deletion frequencies in G1, because it does not hold fragments together; but in the S phase, the excised fragments and the DSB ends are “stapled” to a sister chromatid (Supplementary Material 1). Cohesin acts on multiple levels to organize DSB repair, including retaining sister chromatids for homologous recombination (HR) and replication fork restart (Wu, Yu, 2012); limiting the mobility of the DSB for a better homology search within confined “repair domains” (Piazza et al., 2021); and amplifying the γH2AX signal by asymmetrically extruding flanking chromatin in the vicinity of Ataxia-telangiectasia mutated (ATM) kinase at the DSB (Arnould et al., 2021).


Supplementary Materials are available in the online version of the paper: https://vavilovj-icg.ru/download/pict-2024-28/appx20.pdf


At the same time, cohesin promotes replication stress by interfering with replication during loop extrusion (Minchell et al., 2020), which complicates the picture even further. Another insight comes from the DIvA (DSB Inducible via AsiSI) U2OS cells. This cell line expresses AsiSI restrictase with attached estrogen receptor ligand-binding domain (Aymard et al., 2014). After induction by 4-hydroxytamoxifen (4OHT), AsiSI translocates into the nucleus and introduces around 100–200 DSBs in annotated genomic loci (Dobbs et al., 2022). Multiple breaks induced with AsiSI tend to cluster together and form special kinds of D-compartments, but cohesin is not required for this process or any other kind of chromatin compartmentalization (Schwarzer et al., 2017; Arnould et al., 2023). Trans-interactions of multiple AsiSI-induced DSBs were also not affected by the Rad21 knockdown, but cohesin was required to reinforce affected TADs locally (Arnould et al., 2023). Thus, the connections between cohesin, chromatin compartmentalization, sister chromatid cohesion, DSB restraining and end joining are highly complex.

The role of condensins, another SMC family of DNA organizers, in DSB repair is still unclear. Their primary function is genome compaction before mitosis and they are mostly not active during interphase, although the complex resides in the nucleus throughout the cell cycle. Recent photobleaching experiments indicated that during interphase condensin II is very efficiently blocked from chromatin by the primary binding partner – the microcephalin protein (Mcph1) (Houlard et al., 2021). Mcph1 plays an important but poorly understood role in DSB repair, such as facilitating HR repair through Rad51 filament stabilization (Wu et al., 2009; Chang et al., 2020). Defects in condensin assembly lead to chromosomal aberrations and sister chromatid interlinks in mitotic chromatin (Wu, Yu, 2012; Baergen et al., 2019). Evidence suggests that yeast condensin cooperates with topoisomerase-II to dissolve DNA knots (Dyson et al., 2021) and condensin II could be directly or indirectly involved in homology-directed repair (Wood et al., 2008).

Does cohesin directly impact the joining of close and distant DSBs? How do TAD features (size, borders, chromatin) influence deletion frequencies? Do condensin complexes play any role in DSB repair? In a series of pilot experiments presented here, we begin to explore some of these glaring questions.

Previously, we obtained and extensively characterized mouse embryonic stem cells (mESCs) with auxin-inducible degron (AID) knock-ins for Rad21 (cohesin) and Smc2 (both condensins I+II). These cells exhibit rapid depletion of the target protein within 1–2 hours of auxin addition (Yunusova et al., 2021). Cas9 activity generates blunt ends at the target sites. Using a pair of gRNA frequently leads to an excision of an intermediate DNA segment, which could lead to deletion or inversion after non-homologous or microhomology-mediated end joining (NHEJ/MMEJ) (Canver et al., 2014;Watry et al., 2020; Li et al., 2021). The cell lines were nucleofected with Cas9 and paired gRNAs, and studied using droplet digital PCR (ddPCR) to detect deletions and inversions in the mESCs population. This approach allowed us to assess the influence of the spatial organization of DNA and chromatin on the joining of two distant DNA ends. Overall, the method demonstrated high efficiency and sensitivity for detecting deletions and inversions. At the same time, the results were somewhat inconsistent, and the method we used might be more challenging than we had anticipated. We discuss its potential and limitations in the following chapters.

## Materials and methods

gRNA design and cloning. We selected two genomic regions to induce paired DSBs: the Ace2 gene locus (ChrX: 162.922.328–162.971.416) and a distinct TAD border that shows strong Ctcf signals in ChIP-seq data for mESCs (Chr5: 49.487.342–49.557.342) (GRCm39). High scoring gRNA sites were chosen using Benchling and Aidit algorithms. The sequences of the optimal gRNAs are listed in the Table. All oligonucleotides used in the study were purchased from DNA-Synthesis (Russia). 100 nt gRNAs were synthesized by the T7 in vitro transcription system from a PCR product amplified from a gRNA vector with the T7-primer (overhang 5′-GTTAATACGACTCACTATAG-20nt(gRNA)-3′) and the reverse primer (see the Table) (HiScribe® T7 High Yield RNA Synthesis Kit, E2040S, protocol for short products). After 4 hours at 37 °C, the reaction volume (20 μl) was diluted to 100 μl and treated with 2 μl (4U) of DNaseI (NEB #M0303) in the corresponding buffer. RNA was purified with Monarch® RNA Cleanup Kit (50 μg) (T2040L) and diluted in 30 μl water to achieve concentrations of 2 μg/μl or higher.

**Table 1.**
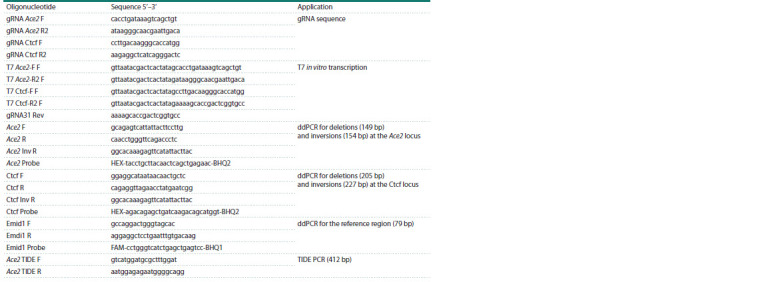
Oligonucleotides used in the study

mESCs nucleofections. Both mESCs auxin degron cell lines were characterized in our laboratory earlier (Rad21- miniIAA7-eGFP, Smc2-miniIAA7-eGFP) (Yunusova et al., 2021). Cells were cultured on plates coated with a 1 % gelatin solution under 2i conditions (1 μM PD, 3 μM CHIR) in DMEM (Thermo Fisher, USA), supplemented with 7.5 % ES FBS (Gibco, USA), 7.5 % KSR (Gibco), 1 mM L-glutamine (Sigma, USA), NEAA (Gibco), 0.1 mM β‐mercaptoethanol, LIF (1000 U/ml, Polygen), and penicillin/streptomycin (100 U/ml each). Upon reaching appropriate confluence (70–80 %), the cells were passaged every two days

**Fig. 2.**
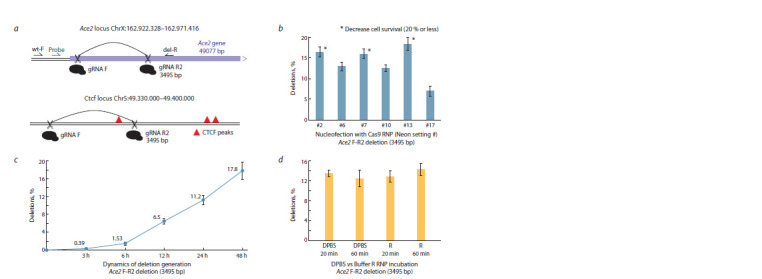
Optimization of Neon conditions for deletion generation a – deletions examined in the study. Primers and the probe for ddPCR are shown for the Ace2 locus; b–d – optimizing mESCs Neon nucleofection conditions with the F-R2 (Ace2) gRNA pair.

**Fig. 3.**
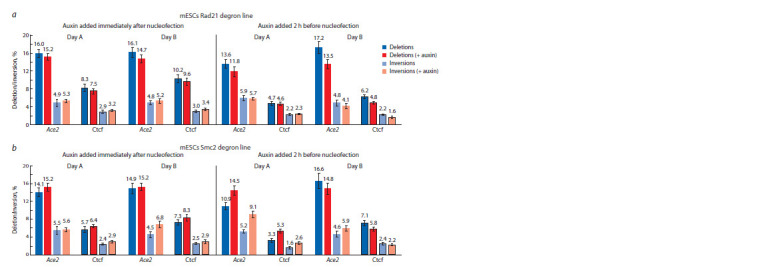
Deletion/inversion frequencies (DIF) for different genomic regions before and after addition of auxin. a – DIF for Ace2 F-R2 and Ctcf F-R2 regions in Rad21 degron line; b – DIF for Ace2 F-R2 and Ctcf F-R2 regions in Smc2 degron line. Data presented as average between three nucleofection replicates and combined SEM. Statistical analysis for mean relative values across four biological experiments is provided in the main text.

Single nucleofection sample consisted of 5 μl Buffer R with 300 000 cells which were mixed with 5 μl of RNP complex diluted in Buffer R in a 10 μl tip. Nucleofections were carried out at Neon preset condition #10 (Pulse voltage 1000, Pulse width 100, Pulse No. 1). Other tested conditions included #2 (1400, 20, 1), #6 (1100, 30, 1), #7 (1200, 30, 1), #13 (1100, 20, 2), #17 (850, 30, 2). The RNP mix consisted of 0.2 μl of concentrated Cas9-NLS protein (30 pmoles) (Biolabmix, Russia) and 2000 ng of each gRNA (1:2 ratio each). We aimed to set two replicates for the technical experiments (see Fig. 2) and three replicates for deletion/inversion frequencies (DIF) measurements (see Fig. 3). Auxin (500 μM of indole-3-acetic acid (IAA)) was added either 2 hours before nucleofection or right after cell plating after nucleofection, and was kept in the culture medium for the whole period. Target protein degradation was confirmed by microscopic analysis of GFP fluorescence loss (Supplementary Material 2). Cells were collected 24 hours after nucleofection. Genomic DNA was isolated from cells using phenol-chloroform extraction

ddPCR assays. Droplet digital PCR (ddPCR) was performed using a QX100 system (Bio-Rad, USA) with primers and probes specific for the Ace2 and Ctcf regions, as well as the reference gene Emid1 (see the Table). ddPCR reactions were set in 20 μl volumes containing 1× ddPCR Supermix for Probes (no dUTP), 900 nM primers and 250 nM probes, and 50 ng genomic DNA. ddPCR reactions for each sample were performed in duplicates. PCR was conducted according to the following program: 95 °C for 10 min, then 45 cycles of 94 °C for 30 s and 58 °C for 1 min, with a ramp rate of 2 °C per second, and a final step at 98 °C for 10 min. The results were analyzed using QuantaSoft 1.7.4 (Bio-Rad). The resulting number was presented as mean +/– combined SEM. For Ace2 DIF calculations, the initial ddPCR results were multiplied by two, because the gene is located at the X chromosome (the mESCs DGES-1 line used in the study has male XY origin). Statistical analysis for relative differences between DIF across the experiments was performed with the Student test (control sample frequencies were set as 1).

TIDE sequencing. We PCR-amplified genomic site corresponding to gRNA sites F for Ace2 (412 bp) from 50 ng of mESCs genomic DNA (samples from Fig. 3) (see the Table). PCR products were purified at 2 % agarose gel and Sanger sequenced using forward primer (reverse primer produced similar estimates in small-scale experiment). Sanger files were compared with wild-type control locus in the TIDE application with mostly default parameters (the start of the alignment window was switched to 91 instead of 100 bp) (http://shinyapps.datacurators.nl/tide/) (Brinkman et al., 2014). Average mutation percentage was calculated for three replicates for each degron.

## Results

Implementing ddPCR assay at the mouse Ace2 locus

**Fig. 1.**
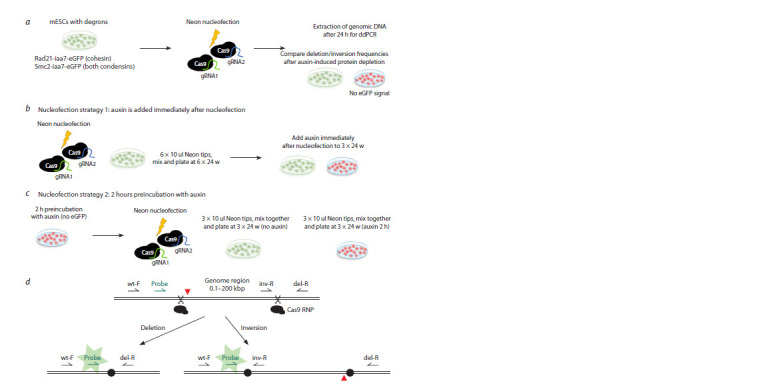
Experimental approach to study deletion/inversion frequencies in mESCs. a – mESCs degrons lines were nucleofected with Cas9 and paired gRNAs; After 24 hours, genomic DNA was extracted and analyzed with ddPCR: we measured relative concentrations of the deletion and inversion alleles against the reference gene (Emid1). Two different nucleofection strategies with respect to auxin addition were tested; b – in the first approach, all cells were mixed together after nucleofection and then split in two sample groups (3+3 × 24 w). Auxin was added immediately after plating to half of the wells; c – in the second strategy, cells were preincubated with auxin for 2 hours and then nucleofected independently of control cells. In both cases, auxin was kept in culture medium for the duration of the experiment (24 hours). Degradation of the Rad21 and Smc2 proteins could be tracked by the loss of eGFP fluorescence (Supplementary Material 2); d – scheme of the droplet digital PCR modification designed to detect genomic rearrangements (ddXR method) (Watry et al., 2020). Induction of paired DNA breaks could lead to excision of the intermediate fragment, resulting in deletion or inversion. The loss or inversion of the fragment allows to efficiently amplify PCR product, activating probe fluorescence.

First, we set out to optimize the Neon nucleofection parameters for mouse embryonic stem cells (mESCs). The outline of a typical experiment is shown in Fig. 1. Following pilot tests, we estimated the average deletion frequency at multiple sites (based on two replicates) across two genomic regions (Fig. 2a). We selected one site (Ace2 F/R2) for Neon optimization. Initially, we tested the nucleofection parameters for wildtype mESCs on a Neon device across 24 basic settings with an EGFP plasmid (data not shown). From this experiment, we identified six conditions demonstrating higher survival rates and GFP fluorescence (conditions #2, 6, 7, 10, 13, 17, 18). Control mESCs were then nucleofected with Cas9 RNP, and the frequencies of deletions were analyzed by droplet digital PCR (ddPCR) (Fig. 2b). We observed a general inverse correlation between cell survival and the efficiency of deletion generation (Fig. 2b; Supplementary Material 3). Consequently, we selected condition #10 for further experiments, since high cell mortality is undesirable in our approach. Overall, the detection of deletion alleles with ddPCR proved to be specific, enabling reliable analysis of genomic DNA from the total mESCs population (Supplementary Materials 4, 5).

To optimize auxin addition time points, we conducted a small experiment to evaluate the timing of the appearance of deletions after nucleofection. It is known that in RNP nucleofection experiments mutations accumulate gradually. In our observations with mESCs, a small percentage of deletions (~2 % of the 48-hour level) appeared already in the first 3 hours after nucleofection (Fig. 2c). After 24 hours, approximately 63 % of deletions from the 48-hour level were observed. Considering the limited survival of mESCs beyond 24 hours without Rad21 or Smc2, this time frame was selected for subsequent experiments with all auxin degron lines. We also performed additional tests of the RNP stability during pre-incubation at 25 °C, revealing that DPBS buffer could effectively substitute the original Buffer R (Neon) without diminishing efficiency (Fig. 2d ).

Deletion/inversion frequencies in mESCs degron lines

Using the established protocol, we measured deletion/inversion frequencies (DIF) at two genomic sites in various chromatin contexts. The Ace2 region was considered a “neutral” region located in the middle of a large TAD and showing no expression in mESCs. Here we focused on the 3495 bp deletion (F-R2) (Fig. 2a). We also analyzed DIF at another genomic site – a strong Ctcf boundary (Chr5:49.487.342–49.557.342) (Fig. 2a; Supplementary Material 6). To account for biological variability, we analyzed two independent experiments that were set with different cell batches and gRNA preparations (Day A, B). We validated these observations with two alternative auxin treatment strategies (Fig. 1b, c). In the first strategy, we first nucleofected the cells, then plated them in six wells and added auxin to half of them. This way, degradation starts simultaneously with Cas9 cutting. In the second strategy, auxin was added 2 hours prior to nucleofection to reliably remove all protein complexes (Fig. 1c). However, this necessitates separate nucleofections for control and treated samples, introducing additional handling variability. Furthermore, protein depletion prior to nucleofection could potentially increase cellular sensitivity to the procedure, possibly affecting ddPCR outcomes.

Surprisingly, we did not observe a Rad21-dependent DIF increase (Fig. 3a). More specifically, we documented a small but reproducible decrease in Ace2 deletion frequencies in all experiments (mean relative decrease across four experiments: –12.1 %, p = 0.0423). Inversion frequencies remained unaffected. It is noteworthy that despite the distinct topological characteristics of the examined regions, there was no visible difference for Rad21-related effects, as the Ctcf region also showed minor and not statistically significant alterations in deletion frequencies (mean relative decrease across four experiments: –10 %, p = 0.109) (Fig. 3a).

Conversely, Smc2 depletion showed a trend towards DIF increase in one of the days (Day A, auxin added 2 hours before nucleofection (Fig. 3)), where it reached +33 % (Ace2 deletions), +75 % (Ace2 inversions), +61 % (Ctcf deletions), +63 % (Ctcf inversion). This effect, however, was not replicated in the subsequent trial (Day B, auxin added 2 hours before nucleofection) (Fig. 3b) upon switching to a different Cas9 batch. Depletion effect on Ace2 deletions was not statistically significant, nor were changes in Ace2 inversion frequencies at a significance level of 0.05 (mean relative increase across four experiments: +39 %, p = 0.088). These discrepancies could be caused by some unaccounted biological factors, such as varying Cas9 batch efficiencies or differences in cell survival post-nucleofection between experiments (see Discussion). The role of the condensin complexes in distant end joining needs additional examinations in the future.

DSB repair efficiency in degron lines

Our objective was to investigate the impact of SMC protein depletion on DSB repair efficiency, particularly at a single DSB site or at closely positioned pairs of DSBs. Previous research indicated that 34 bp deletions are repaired differently from larger 3200 bp deletions in Rad21-deficient cells (Gelot et al., 2016). One of the drawbacks of the ddPCR method is its inability to detect small deletions, due to interference with the wild-type locus amplification. The authors of the ddXR method recommend digesting genomic DNA to selectively eliminate wild-type genomic loci from ddPCR amplification. With this trick, they were able to amplify deletions as small as 91 bp (Watry et al., 2020).

**Fig. 4.**
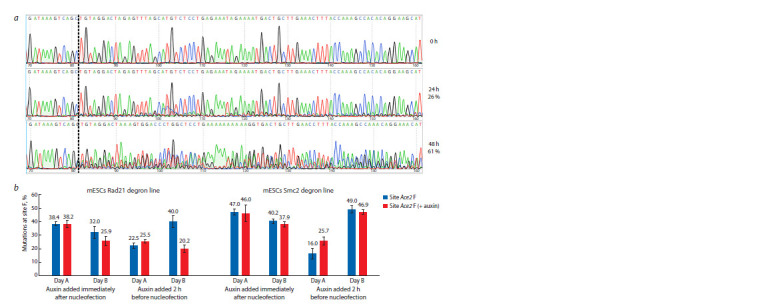
DSB repair efficiency at a single site Ace2 F, measured by TIDE. a – demonstration of Sanger data for the control unedited locus and the mutated locus in the cells from Fig. 2, d. Cut site is marked with a dotted line. Editing efficiency measured with TIDE is shown as %; b – frequencies of site modifications in various degron lines from Fig. 3. Data shown as average and SEM.

We managed to apply restriction to a region of 192 bp at the Ace2 locus (Supplementary Material 7), although attempts to apply it to other short deletions were less successful (data not shown). Given these limitations, the ddPCR method was deemed unsuitable for analyzing 34 bp deletions. Instead, to screen how SMC-protein depletion affects DSB repair at a single end we utilized the Tracking of Indels by DEcomposition (TIDE) method (Brinkman et al., 2014), a straightforward approach based on Sanger sequencing of the break site. This method facilitates the demultiplexing and calculation of Cas9 cut signatures at the break, thereby estimating DSB repair efficiency as a percentage of mutant alleles. Estimating indel mutation signatures at the break site serves not only as an indicator of Cas9 activity but also as a measure of nucleofection efficiency (Fig. 4). We PCR amplified and sequenced regions at the Cas9 target site for the Ace2 F gRNA (Fig. 2a) (the same samples analyzed with ddPCR (Fig. 3)).

For the Smc2 experiments, we did not detect any significant differences in editing efficiency. The slight decrease in efficiency post-auxin treatment was counterbalanced by an increase in DIF (since deletion/inversion events eliminate Ace2 F sites from PCR amplification in TIDE analysis) (Fig. 4b). For Rad21 depletions, a decrease in editing efficiencies was noted, potentially reflecting increased cell vulnerability under high RNP loads in the absence of Rad21. Notably, one experimental condition (Rad21/Smc2, Day A, auxin added 2 h before nucleofection) exhibited a 2-fold reduction in editing efficiencies. In this scenario, auxin addition paradoxically enhanced Cas9 editing for both degron lines (Fig. 4b), yet DIF were impacted differently in Rad21 and Smc2 lines (Fig. 3). This suggests that at lower editing efficiencies (RNP load), cells might respond differently to protein depletion. For example, Smc2 depletion could permeabilize cells for nucleofection, possibly due to a cell cycle shift or chromosome decondensation. We plan to perform nucleofections with various RNP concentrations in the future to verify this effect.

## Discussion

We have performed a series of experiments with auxin degradation and CRISPR/Cas9-induced DSBs using a collection of mESCs with the SMC degrons. mESCs represent an interesting object for studying DNA repair. For instance, mESCs mostly rely on HR to preserve genome stability (Choi et al., 2017) and have different end-joining mechanisms based on specialized polymerases (Schimmel et al., 2017). Since mESCs are difficult to edit with lipofection, we adapted a protocol to generate deletions with Neon nucleofections. This method, in conjunction with ddPCR, demonstrated high efficiency and sensitivity in detecting deletions and inversions, with an average modification rate of 60 % for the Ace2 locus after Neon nucleofection (TIDE at the F site + deletions + insertions) (Fig. 3, 4). This level of editing is notable compared to plasmid transfection outcomes without selection. However, we encountered significant variability in deletion/ inversion frequencies (DIF) across experiments, highlighting the influence of numerous biological factors on experimental outcomes.

Cas9, a crucial component in our experiments, can significantly impact DSB repair dynamics. Variations in the Cas9:gRNA ratio can dramatically alter editing outcomes (Chenouard et al., 2023), with repair processes potentially delayed up to 20 hours due to persistent Cas9-DNA binding (Kim et al., 2014; Brinkman et al., 2018). Furthermore, Cas9 retention at break sites can modify blunt ends into 3′-overhang trimmed ends (Stephenson et al., 2018; Jones et al., 2021), necessitating different polymerases for non-homologous endjoining. Variability was also observed between different lots of Cas9-NLS (Biolabmix) even at identical molar concentrations. To account for all these issues, we performed experiments with two strategies of auxin addition and set three nucleofection replicates. We also performed two biological replicates with different mESCs batches, gRNA preps and Neon tips. From our experience, such experiments require very careful examination of the optimal experimental conditions, especially when the gene of interest has strong pleiotropic effects on cell homeostasis

Our timing analysis indicated that cells accumulate 70 % of deletions within 24 hours, and only 2 % in the first 3 hours, suggesting that auxin could be added within a 0–3 hour window after nucleofection without significantly compromising deletion generation. Furthermore, we confirmed that DPBS incubation does not compromise RNP activity, providing a viable alternative to Buffer R. Notably, immediate post-nucleofection auxin addition exhibited lesser variability com- pared to a 2-hour pre-incubation strategy (Fig. 3), demonstrating the feasibility of its use in future setups due to its uniform experimental conditions

Analysis of data on the frequencies of deletions and inversions for various mESCs clones with degrons allowed us to draw the following conclusions. We expected that Rad21 depletion will cause elevated rates of deletions and inversions due to unconstrained movement of the DSB ends, as it was reported by another group. In their report, there was a 30 % increase in the amount of cells with a 3 kb deletion (Gelot et al., 2016) after Rad21 siRNA knockdown. Some other reports using cytogenetic and microscopic analysis also suggested that Rad21 knockdown provokes DNA rearrangements (Wu, Yu, 2012). So far, we have not found any significant stimulatory effects of Rad21 depletion on DIF (Fig. 3). Given that the authors of the initial report (Gelot et al., 2016) worked with a different experimental setting (plasmid transfection with inducible I-SceI, siRNA Rad21 knockdown, SV40- transformed GM639 human WT fibroblasts) and had an alternative detection strategy, our results may reflect differences between the experimental systems. In our setting, the protein was removed almost completely after 2 hours (Yunusova et al., 2021) and Cas9 RNP was active from the beginning (see timings, Fig. 2c). Also, mESCs are more sensitive to DNA damage and may react to DSB differently than immortalized fibroblasts (Choi et al., 2017).

It is possible that the absence of Rad21 sensibilizes cells to DNA damage resulting in a decreased opportunity for distant end-joining events to happen, hiding the stimulatory effect. This would lead to a lower amount of TIDE signal, as we see in our data (Fig. 4b). However, this does not explain why inversion frequencies are not negatively affected (Fig. 3). In theory, the effect of Rad21 degradation may be more noticeable for extremely distant DSBs, such as a 26 kbp deletion that we plan to analyze in the future (Supplementary Material 6). Correlation between topology and DSB is another long-standing question. In our case, deletion over the Ctcf site at the TAD border was not noticeably affected by cohesin depletion.

Unexpectedly, our findings hint at a significant role of Smc2 depletion in promoting genomic rearrangements, although data variability necessitates further investigation. Condensins are not directly involved in DNA repair, but could affect it via side effects (defects in chromosome segregation, chromatin decondensation in G2/M). Cell cycle is an important determinant of a DSB repair outcome. It is well known that G1 DSBs are repaired with slower kinetics (Arnould et al., 2023). Synchronization of human fibroblasts in the G1 phase showed no end-joining stimulation from Rad21 knockdown (Gelot et al., 2016). We and others analyzed cell cycles in mESCs with Rad21 and Smc2 depletion and found that after 6 hours they accumulate in the G2/M phase (manuscript under preparation). Judging from these data, Rad21 and Smc2 clones have the same cell cycle profile. Thus, cell cycle shift alone would not explain the difference between Rad21 and Smc2 depletion effects. In this study, we could only work with an unsynchronized mESCs population. Synchronization of mESCs is very challenging and imposes additional cell lethality making this approach unsuitable for our goal.

We plan to expand our investigations with the repertoire of deletions at other genomic regions with interesting topological organization. We will also try other improvements, such as NGS sequencing with Unique Molecular Identifiers (UMIs) for Cas9 target sites to account for editing efficiency. In the future, we will also extend our findings to simpler, synchronizable human cell lines such as HAP1 and HCT116, which also harbor Rad21/Smc2 degrons, to further dissect these complex dynamics.

## Conclusion

Cohesin facilitates genome stability by limiting DNA movements during replication. By this logic, supported by experimental data, the frequencies of deletion between paired distant breaks will increase after cohesin removal. We could not reproduce these findings in the Rads21 auxin-degron cell line as we did not see an increase in deletion or inversion frequencies. This may reflect differences between experimental systems. Both Rad21 and Smc2 degron studies will require more iterations to account for biological variability.

## Conflict of interest

The authors declare no conflict of interest.
